# A modified primer-pair designed for the molecular sex identification of black-legged Kittiwake (Laridae: *Rissa tridactyla*)

**DOI:** 10.1007/s12686-025-01400-x

**Published:** 2025-07-02

**Authors:** Chloe P. Cargill, George Harrand, Signe Christensen-Dalsgaard, Alexey V. Ezhov, Ruben C. Fijn, Maria V. Gavrilo, Mark A. Newell, Carmel McDougall

**Affiliations:** 1 University of Aberdeen (School of Biology), Aberdeen, United Kingdom; 2 University of St Andrews (Scottish Oceans Institute, Instituid Chuantan na h-Alba), St Andrews, United Kingdom; 3 Norwegian Institute for Nature Research (NINA), Trondheim, Norway; 4 Kandalaksha State Nature Reserve, Kandalaksha, Russia; 5 Waardenburg Ecology, Department of Bird Ecology, Culemborg, The Netherlands; 6 Association Maritime Heritage: Explore and Sustain, Saint Petersburg, Russia; 7 Arctic and Antarctic Research Institute, Saint Petersburg, Russia; 8 Centre for Hydrology & Ecology, Edinburgh, United Kingdom

**Keywords:** Primer, Charadriiformes, Sexing, Feather, CHD1

## Abstract

In studies of avian populations accurate sex identification facilitates the study of sex-linked behaviour, ecology, social structure, decision-making, and life histories. To improve molecular sex identification protocols for the black-legged kittiwake (Laridae: *Rissa tridactyla*, hereafter ‘kittiwake’), primers previously developed for other bird species were realigned against the kittiwake CHD1 Z and W chromosomes to generate a species-specific primer-pair. Amplicons of 602 bp and 375 bp were produced using typical PCR and gel electrophoresis procedures. The modified primer-pair was validated on a test sample of 138 kittiwakes from four Atlantic breeding colonies sexed a priori using head-bill length.

The black-legged kittiwake (*Rissa tridactyla*), hereafter referred to as ‘kittiwake’, is a seabird from the Laridae family that breeds in the arctic and boreal zones of the Northern Hemisphere. Given its conservation status – ‘Vulnerable, declining’ on the IUCN Red List of Threatened Species (Birdlife International 2019) – sex-linked data are required for improved conservation and management (see Horswill and Robinson 2015). Morphological sexing commonly uses the sum of the lengths of the head and bill (‘head-bill length’) (Jodice et al. 2000; Coulson 2009), which are recorded during ringing. Molecular sex identification is useful when morphometrics are ambiguous (see Coulson 2009), unavailable (i.e., studies using dropped/moulted feathers), or where the focal age-class does not show sexual dimorphism (fledglings). Like all birds, kittiwakes possess the ZW sex-determination system: heterozygous female with genotype ZW; homozygous ZZ male. Avian molecular sex identification commonly utilises the Chromodomain Helicase DNA-binding Protein 1 (CHD1) genes, which exist as very similar copies on the Z and W chromosomes, with different intron sizes (Fridolfson and Ellegren 2012). Sex identification of kittiwakes has previously utilized primer-pairs developed variously for parrots, fowl and passerines (summarised in Fig. 1A) either in combination (e.g., Merkling et al. 1999) or alongside behavioural observations and morphometrics (e.g., Jodice et al. 2000). The most reliable primer-pair (P2F/P8R) generates amplicons with only a 10 bp difference between Z and W variants for the kittiwake, making scoring difficult (Merkling et al. 2012).

Here, a kittiwake-specific primer-pair for molecular sex identification is presented. The GenBank nucleotide database was searched for *R. tridactyla* CHD1 mRNA sequences (query ‘Rissa tridactyla[Organism] CHD1’). Resulting sequences were used in a BLASTn search against a recently published high quality chromosome-level reference genome for the Pacific subspecies (*R. t. pollicaris*) (Sozzoni et al. 2023; GenBank accession GCA_028500815.1). BLAST searches produced high-scoring (E-value 0.0) matches on both the Z and W chromosomes. Genomic regions covering the BLAST hits were downloaded and aligned with the CHD1 mRNA sequences to reveal intron/exon boundaries using AliView (Larsson 2014). The CHD1 primers 2550 F and MSZ1R, which are less reliable than P2F/P8R in *R. tridactyla* but produce Z and W variant amplicons with greater size difference, were realigned and adjusted, adding degeneracy where mismatches between the Z and W chromosomes occurred (Fig. 1A). The resulting primer-pair, named RT_2550F/RT_MSZ1R (Fig. 1A), generates a 602 bp Z amplicon and a 375 bp W amplicon (Fig. 1B-C).

**Fig. 1:**
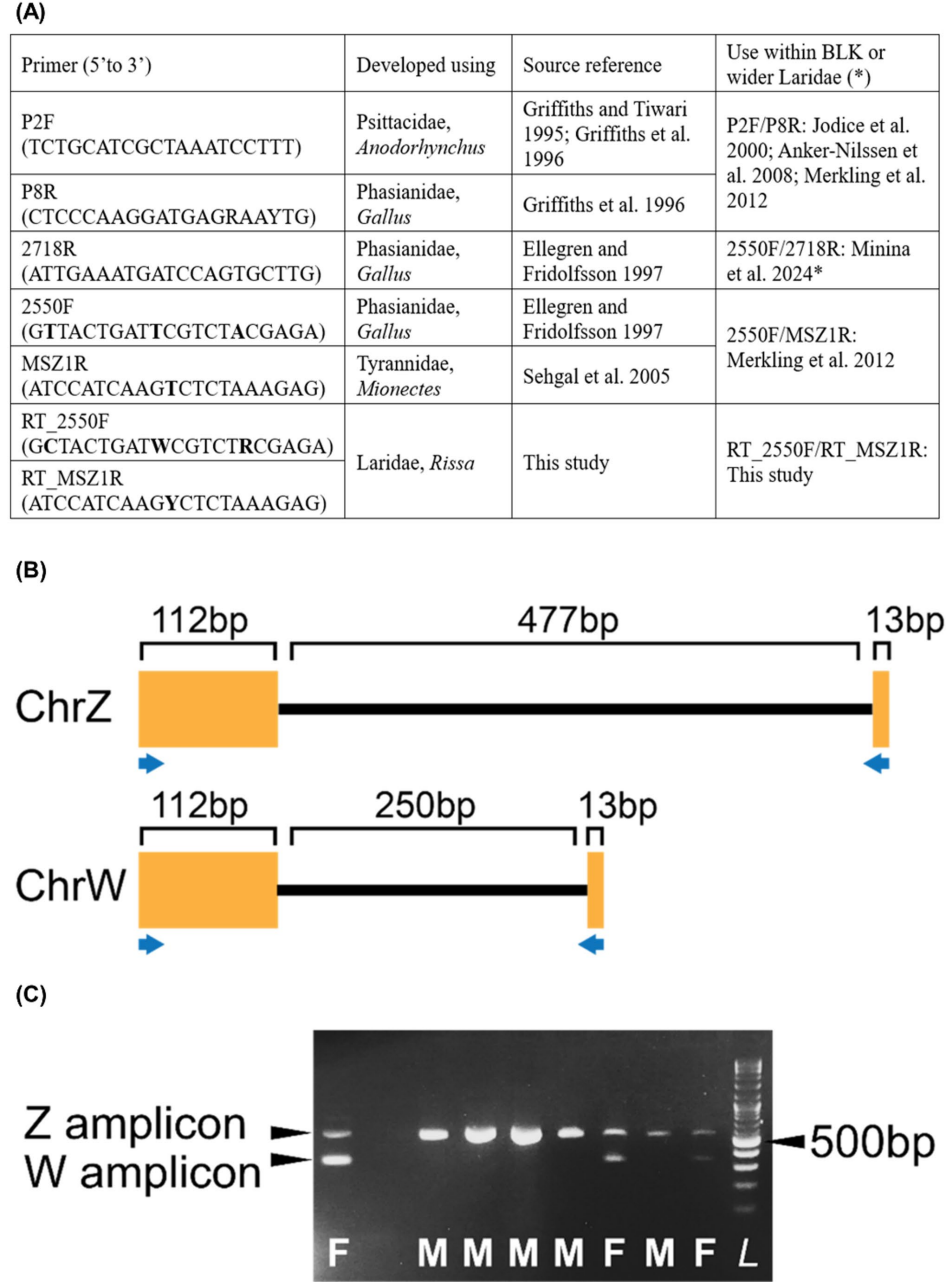
**(A)** Primer sequences and primer-pairs used in the sex identification of black-legged kittiwake (BLK) or close relatives, and their source Family and species. Modifications to the 2550 F/MSZ1R primers are in **bold**. **(B)** Schematic of partial *R. tridactyla* CHD1 genes from the Z and W chromosomes demonstrating the difference in intron length (bold black line). The reverse primer spans an intron/exon boundary. **(C)** Visualisation of 5 µL of PCR product from seven kittiwakes on a 1% TBE gel. Amplicons are observed at 602 bp (Z) and 375 bp (W), shown against a 1 Kb + ladder (*L*). Male kittiwakes (M) are ZZ and females (F) are ZW

PCR conditions for optimisation comprised denaturation at 95^o^C for 30 s, followed by 35 cycles of 95^o^C denaturation for 30 s, annealing across a thermogradient (from 49.2^o^C to 52.1^o^C) for 30 s, 68^o^C elongation for 1 min, and a final elongation step at 68^o^C for 5 min. Clear amplicons were derived across the thermogradient tested at template DNA concentrations ranging from less than 0.0025 ng/µL to over 120 ng/µL. 25 µL PCR reaction mixtures contained 2 µL of template genomic DNA, 0.125 µL of Taq DNA Polymerase (New England Biolabs, NEB), 0.5 µL of Standard Reaction Buffer (NEB), 0.5 µL of dNTP mix (NEB), 18.875 µL of sterile ddH_2_O and 0.5 µL of each primer.

The modified primer-pair was tested using opportunistically collected feathers of 138 kittiwakes sexed a priori from head-bill length (Fig. 2A), sampled across the breeding range of the Atlantic subspecies (*R. t. tridactyla*) (Fig. 2B). Genetic cross-contamination was prevented by sterilizing the cutting surface and tools with 0.1% bleach solution and rinsing with sterile water between samples.

**Fig. 2:**
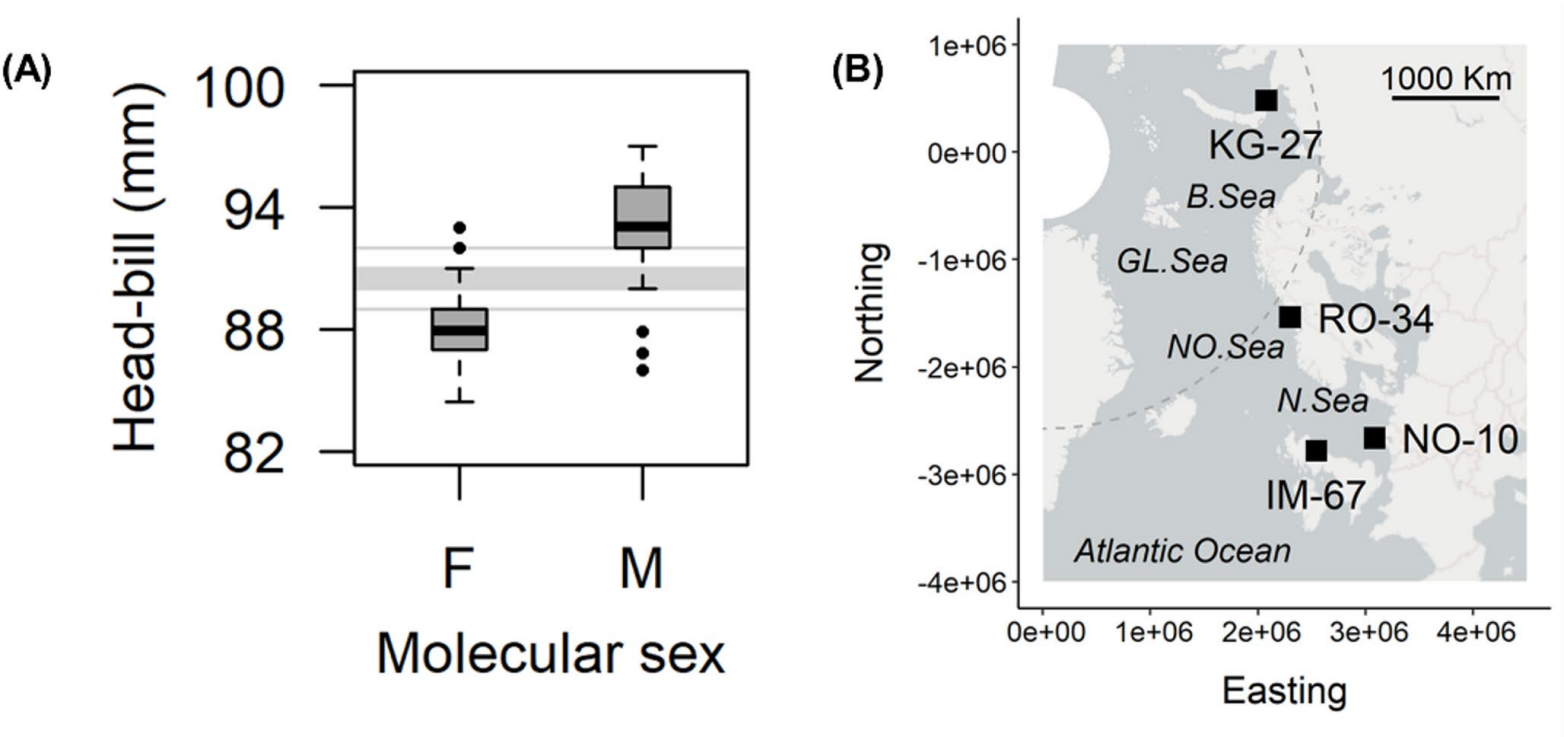
**(A)** Testing molecular sex identification in black-legged kittiwakes (*n* = 138) using primer-pair RT_2550F/RT_MSZ1R. A head-bill length below 89 mm (lower grey line) is expected to be female (F); above 92 mm (upper grey line) male (M); between 90–91 mm (shaded region) equally male or female (after Coulson 2009). **(B)** Spatial distribution of kittiwake colonies (black squares) and sample sizes. Abbreviations: KG Kara Gate, Russia; RO Rørvik, Norway; NO offshore Platform L7B, The Netherlands; IM Isle of May, Scotland; *B. Sea* Barents Sea; *GL. Sea* Greenland Sea; *NO. Sea* Norwegian Sea; *N. Sea* North Sea. Dashed grey line: Arctic Circle southern extent. Produced in R (R Core Team 2023)

Molecular results agreed with a priori sex in 133 of the 138 kittiwakes tested. Likely explanations for the incongruence of molecular and morphometric data observed in five individuals (from Norway and Scotland) are, inaccurate head-bill length data, true morphometric outliers (i.e., smaller males and larger females than expected under percentage frequency distribution of head-bill length assumed here, see Coulson 2009), or failure of the target region on the W chromosome to amplify (for the ‘small males’). Overall, we provide a validated species-specific tool for the further study of sex-linked kittiwake behaviour, ecology, social structure, decision-making, and life histories. Further, given the general versatility of existing primer-pairs for avian molecular sex identification (Fig. 1A and e.g., Bond et al. 2016), these updated primers and the methods described are likely to be highly applicable within future studies of other *Rissa* spp., and the wider Laridae and Charadriidae.

## Data Availability

Primer sequences are provided within the manuscript. The morphometric data used and the molecular sex data generated within this study are available from the corresponding author on reasonable request.
